# The Omicron Wave in Tunisia: Dynamic, Diversity, and Phylogenetic Analyses

**DOI:** 10.3390/microorganisms13092162

**Published:** 2025-09-17

**Authors:** Yasmine Chaari, Sondes Haddad-Boubaker, Haifa Khemiri, Wasfi Fares, Anissa Chouikha, Cesare Camma, Alessio Lorusso, Hanen Smaoui, Khaoula Meftah, Ouafa Kallala, Abdelhalim Trabelsi, Amel Chtourou, Awatef Taktak, Olfa Bahri, Manel Hamdoun, Yassine Chaabouni, Henda Touzi, Amel Sadraoui, Zina Meddeb, Nissaf Ben Alaya, Mariem Gdoura, Henda Triki

**Affiliations:** 1 Faculty of Pharmacy of Monastir, University of Monastir, Monastir 5000, Tunisia; chaariyasmine12@gmail.com; 2 Laboratory of Clinical Virology, WHO Regional Reference Laboratory for Poliomyelitis and Measles for in the Eastern Mediterranean Region, Institut Pasteur de Tunis, University of Tunis El Manar, 13 10 place Pasteur, BP74 1002 le Belvédère, Tunis 1002, Tunisia; haifa.khemiri@pasteur.utm.tn (H.K.); fwasfi@yahoo.fr (W.F.); chouikhaanissa@gmail.com (A.C.); touzihenda@yahoo.fr (H.T.); amelsedraoui1@gmail.com (A.S.); zinamedeb@gmail.com (Z.M.); mariemgdoura@gmail.com (M.G.); henda.triki@pasteur.rns.tn (H.T.); 3 Research Laboratory “Viruses, Vectors, and Hosts” (LR20IPT02), Institut Pasteur de Tunis, University of Tunis El Manar, Tunis 1002, Tunisia; 4 Clinical Investigation Center (CIC), Institute Pasteur de Tunis, University of Tunis El Manar, Tunis 1002, Tunisia; 5 Department of Virology, Istituto Zooprofilattico Sperimentale dell’Abruzzo e del Molise, 64100 Teramo, Italy; c.camma@izs.it (C.C.); a.lorusso@izs.it (A.L.); 6 Department of Microbiology, Faculty of Medicine of Tunis, University of Tunis El Manar, Tunis 1007, Tunisia; hanen.smaoui@gmail.com (H.S.); meftahkhaoula@gmail.com (K.M.); olfa.bahri@fmt.utm.tn (O.B.); manel.hamdoun@fmt.utm.tn (M.H.); nissafba@yahoo.fr (N.B.A.); 7 Laboratory of Microbiology, Microbiology of Children and Immunocompromised, Faculty of Medicine of Tunis, University of Tunis El Manar, Tunis 1006, Tunisia; 8 Laboratory of Microbiology, Bechir Hamza Children’s Hospital, Tunis 1006, Tunisia; 9 Laboratory of Microbiology, Sahloul Hospital of Sousse, Sousse 4054, Tunisia; ouafa.kallala@gmail.com (O.K.); abdelhalim.trabelsi@gmail.com (A.T.); 10 Laboratory of Microbiology, Habib Bourguiba University Hospital, Rue El Ferdaous, Sfax 3003, Tunisia; amel11.doc@gmail.com (A.C.); awateftaktak@yahoo.fr (A.T.); 11 Laboratory of Microbiology and Biochemistry, Aziza Othmana Hospital, Tunis 1068, Tunisia; 12 Laboratory of Medical Biology, Ibn El Jazzar Hospital, Kairouan 3100, Tunisia; chaabouniyassine@yahoo.fr; 13 National Observatory of New and Emergent Diseases, Ministry of Health, Tunis 1002, Tunisia; 14 Department of Virology, Faculty of Pharmacy of Monastir, University of Monastir, Monastir 5000, Tunisia

**Keywords:** SARS-CoV-2, omicron variant, recombinants (XBB; XDK; XDQ), phylogenetic analysis, North Africa

## Abstract

The SARS-CoV-2 Omicron variant has exhibited a rapid progression around the world, but its molecular insights in North Africa remain understudied. This study characterizes the genetic diversity, dynamics, and evolutionary trends of the Omicron variant in Tunisia over a 33-month period (December 2021–August 2024). In total, 928 high-quality whole-genome sequences were considered in this study, of which 559 were retrieved from the GISAID database and 369 were generated in our laboratory. Phylogenetic analysis of the dominant subvariants (BA.1, BA.2, and BA.5) was performed using IQ-TREE. BA.2 was the predominant subvariant (38%), followed by BA.1 (24.0%), Omicron recombinants (19%), and BA.5 (18%). BA.2 diversified into JN, KP, and BN sub-lineages. Recombinants were dominated by XBB (98.8%), primarily including EG.4, XBB.1.5, and XBB.2.3.11, with rare detection of XDK and XDQ. Phylogenetic analysis revealed local clusters in BA.1, BA.2, and BA.5 alongside imported strains. Tunisia’s Omicron wave was mainly driven by BA.2 and its recombinants, with evidence of localized viral evolution and sporadic introductions. The detection of rare recombinants underlines the importance of integrating regional genomic surveillance with epidemiological data in order to help guide future public health strategies.

## 1. Introduction

After its emergence in late 2019 in Wuhan, China, Severe Acute Respiratory Syndrome Coronavirus 2 (SARS-CoV-2) spread quickly around the globe; it took only a few months for a pandemic state to be issued by the World Health Organization (WHO) on 11 March 2020 [1]. As of 22 September 2024, a total of 776,386,491 confirmed Coronavirus Disease (COVID-19) cases have been reported globally, with over 7 million deaths worldwide [2].

SARS-CoV-2 belongs to the *Coronaviridae* family, *Orthocoronavirinae* subfamily, *Betacoronavirus* genus, and *Sarbecovirus* subgenus. It is an enveloped single-stranded, positive-sense RNA virus with a spherical shape that contains spike-like projections on its surface, giving it a crown-like appearance [3]. Throughout the pandemic, the SARS-CoV-2 virus has been evolving continuously with a mean substitution rate of 0.6–1.6 10^−3^ substitutions per site per year, depending on the variant [4]. SARS-CoV-2 variants were classified by the WHO into three groups: Variants of Concern (VOCs), Variants of Interest (VOIs), and Variants Under Monitoring (VUMs) [5]. To track global SARS-CoV-2 lineage transmission, a nomenclature system known as the Pango lineage nomenclature was developed by Rambaut (2020) [6]. The major VOCs identified so far are Alpha (B.1.1.7), Beta (B.1.351), Gamma (P.1), Delta (B.1.617.2), and Omicron (B.1.1.529), each with distinct protein sequences and varying biological characteristics [7].

The Omicron variant was first reported on 24 November 2021, in Gauteng, South Africa, and was classified as a VOC just two days later [7]. By 16 December 2021, it had spread to 87 countries and was associated with a sharp surge in COVID-19 cases [7]. Compared to earlier VOCs, Omicron accumulated a significantly higher number of mutations [8]. Notably, the triple mutations in the furin-like cleavage site, H655Y, N679K, and P681H, have been linked to its enhanced transmissibility [9], contributing to a faster and more widespread transmission. Additionally, the Omicron variant exhibits a 2-to-2.5-fold greater binding affinity to the angiotensin-converting enzyme 2 (ACE2) receptor than the original SARS-CoV-2 strain, largely due to the T478K, Q493K, and Q498R mutations within its receptor-binding domain (RBD) [10]. Furthermore, Omicron has demonstrated a remarkable ability to evade most virus-neutralizing antibodies, whether induced by vaccination or prior infection with other variants [11].

According to the report published by WHO in April 2022, the five major Omicron subvariants are BA.1, BA.2, BA.3, BA.4, and BA.5 [7].

During the pandemic, the ongoing emergence of these SARS-CoV-2 variants has facilitated co-infection, increasing the likelihood of genetic recombination, a phenomenon achieved when two different strains infect the same cell. This led to the emergence of recombinant subvariants, not only between distinct lineages, such as the Deltacron (XD, XF, and XE), but also within the same lineage [12]. A prominent example is the Omicron XBB recombinant, which stemmed from two second-generation Omicron BA.2 sub-lineages [12].

Despite the global interest in Omicron’s evolution, data in Tunisia and North Africa remain limited. Chouikha et al. and Haddad-Boubaker et al. offered a genetic analysis of SARS-CoV-2 evolution in Tunisia over the first 17 months of the pandemic and the Delta wave in Tunisia, respectively [13,14]. In a subsequent study of the Tunisian pediatric population, Khemiri et al. reported Delta as the predominant variant (39.8%) between April 2020 and February 2022, followed by Omicron (24.2%) and Alpha (13.9%) [15].

The present study aims to analyze the Omicron wave epidemiology and its genetic features in Tunisia, to identify predominant subvariants, and to assess their phylogenetic relationships with Omicron strains reported from other regions of the world.

## 2. Materials and Methods

### 2.1. Ethical Statement

This study was approved by the Bio-Medical Ethics Committee of the Pasteur Institute of Tunis, Tunisia (2020/14/I/LR16IPT/V1), on 24 November 2020. It was performed under ethical standards according to the 1964 Declaration of Helsinki and its later amendments. All samples were investigated after de-identification with respect to patient anonymity.

### 2.2. Samples and Viral Genome Sequencing

A total of 369 sequences were obtained and submitted at the laboratory of Clinical Virology of the Pasteur Institute of Tunis. These sequences originated from SARS-CoV-2 positive nasopharyngeal swabs collected between 1 January 2022 and 13 February 2023, corresponding to the Omicron circulation period in Tunisia. Samples were gathered at the Pasteur Institute of Tunis along with various laboratories across Tunisia. All contributors are detailed in Supplementary Table S1. They were transported under refrigerated conditions and processed within 24 h with genome extraction and real-time PCR detection as previously described [14].

Full-genome sequencing was performed using NGS platforms at two centers: the Pasteur Institute of Tunis (Illumina COVID Seq 1000 (San Diego, CA, USA); 339 sequences) and the collaborating Istituto Zooprofilattico Sperimentale dell’Abruzzo e del Molise, Teramo, Italy (NextSeq 500; *n* = 30). Viral RNA extraction, RT-PCR genotyping, amplification, and sequencing were performed using either commercial kits or in-house protocols as previously described [14,15]. Consensus genome sequences in FASTA format were uploaded to Pangolin (v4.3) for lineage assignment (https://cov-lineages.org (accessed on 20 March 2025)). Only sequences covering at least 85% of the genome were selected for sub-lineage classification. Nextclade v3.16.0 (https://clades.nextstrain.org/ (accessed on 19 March 2025)) was used to assign clades and assess diversity, amino acid changes, and mutation profiles. The identification of SARS-CoV-2 lineages and sub-lineages was performed on the consensus sequences in FASTA format using Pangolin (version 4.3) (https://cov-lineages.org/pangolin.html (accessed on 10 March 2025)).

### 2.3. Viral Sequences

A total of 1194 were retrieved from the Global Initiative on Sharing All Influenza Data (GISAID) database [16] and from GenBank (NCBI) [17]. They included Omicron Tunisian genomic sequences (*n* = 559) and other worldwide sequences (*n* = 635).

Concerning the Omicron Tunisian genomic sequences, they were derived from samples collected between December 2021 and August 2024; the selection criteria included sequences from “Africa/Tunisia” and the variant criteria were “Former VOC Omicron GRA (B.1.1.529 + BA.*)”. Samples originated from 21 out of 24 Tunisian governorates as detailed in Supplementary Table S1. As for the worldwide sequences, they were randomly distributed as follows: Africa (*n* = 12), Asia (*n* = 121), Europe (*n* = 242), North America (*n* = 198), Oceania (*n* = 9), and South America (*n* = 53), as provided in Supplementary Table S2. To ensure the accuracy and reliability of the genomic analysis, only sequences with less than 10% ambiguous nucleotide positions were considered.

### 2.4. Phylogenetic Analyses

Phylogenetic analysis concerned major Omicron subvariants: BA.1, BA.2, and BA.5. The selected dataset comprised 657 high-quality Omicron sequences (BA.1, BA.2, and BA.5) from Tunisia and 635 worldwide sequences. Sequence alignment was conducted using the MAFFT online platform (version 7) (https://mafft.cbrc.jp/alignment/software/ (accessed on 30 March 2025) [18] with default settings. Maximum Likelihood phylogenetic trees were constructed with IQ-TREE multicore software (v1.6.12) (http://iqtree.cibiv.univie.ac.at/ (accessed on 7 April 2025) [19] employing 1000 bootstrap replicates to evaluate the robustness of the tree topology. The resulting phylogenies were visualized and annotated using FigTree (version 1.4.4) (http://tree.bio.ed.ac.uk/software/figtree/ (accessed on 8 April 2025) [20].

## 3. Results

### 3.1. Epidemiological Features of Collected Samples

This study included 928 Tunisian sequences obtained from 414 males and 514 females, with a sex ratio of 1.24. The ages of the individuals ranged from 18 days to 98 years, with a mean age of 42.82 years and a median age of 44 years. The highest proportion of cases was observed among individuals aged 18–44 years (30.8%), followed by those aged 45–64 years (28.5%) and ≥65 years (18.0%). Pediatric cases were less common, with children aged 1 month to 9 years representing 9.3% of the cohort and adolescents aged 10–17 years accounting for 7.5%.

### 3.2. Variant Assignment

**Figure 1:**
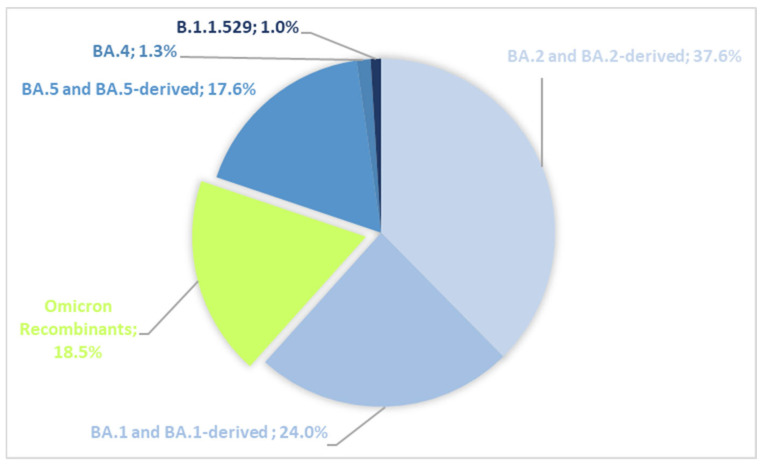
Omicron variant assignment and distribution among the Tunisian general population.

Among the 928 analyzed Tunisian Omicron sequences, 9 were designated as parental lineage B.1.1.529, 747 belonged to 11 distinct Omicron subvariants, and 172 were recombinants. Omicron subvariants included BA.1.* (*n* = 223), BA.2.* (*n* = 310), BA.5.* (*n* = 131), and BA.4 (*n* = 12). The main BA.2-derived sub-lineages were JN.* (*n* = 32), BN.* (*n* = 2), and KP.1.* (*n* = 2), along with CH.1.1, CM.8.1, and LA.1 (*n* = 1 each). As for BA.5-derived sub-lineages, BQ.* (*n* = 22) and BE* (*n* = 10) were identified. Recombinants were predominantly XBB (*n* = 170 out of 172) (Figure 1).

**Figure 2:**
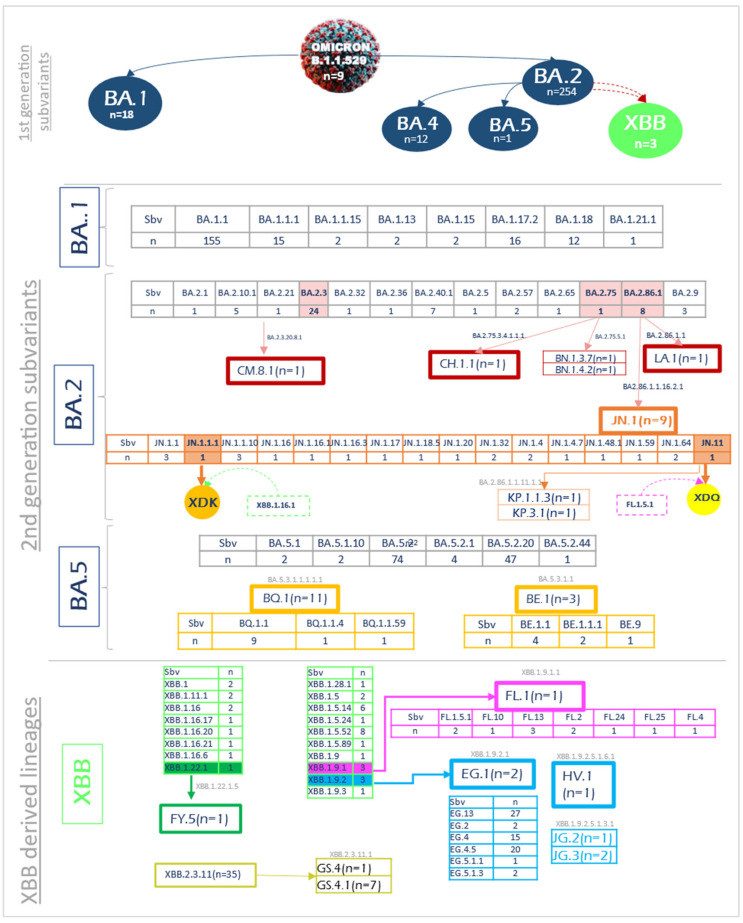
Omicron subvariant diversity and molecular evolution among the Tunisian general population. Sbv subvariant; n: number.

Considering second-generation subvariants, a total of 107 were identified (Figure 2). Within BA.1, the detected lineages included BA.1.1, BA.1.1.15, BA.1.1.18, BA.1.1.20, BA.1.1.21, and BA.1.1.11. BA.2 comprised BA.2.1, BA.2.3, BA.2.5, BA.2.7, BA.2.10.1, BA.2.10.2, BA.2.12, BA.2.27, BA.2.57, BA.2.75, BA.2.86.1, and BA.2.86.3, along with derived lineages such as JN.1.*, JN.11, LA.1, BN.1.*, CM.8.1, CH.1.1, and KP.*. As for BA.5, its sub-lineages were BA.5.1, BA.5.1.2, BA.5.2.20, BA.5.2.44, BE.1.*, BE.9, and BQ.1.*. The XBB lineage encompassed XBB.2.3.11, XBB.1.5.*, XBB.1.9.*, XBB.1.11, XBB.1.16, XBB.1.22, and XBB.1.28, as well as EG.*, FY.5, FL.*, JG.*, HV.1, and GS.4.*. Additionally, XDQ and XDK were detected (Figure 2).

### 3.3. Distribution Timeline of the Omicron SARS-CoV-2 Subvariants

**Figure 3:**
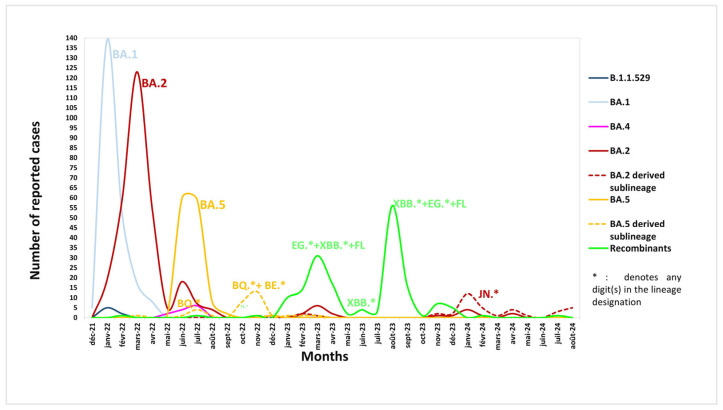
Omicron subvariant evolution among the Tunisian general population during the study period.

The first Omicron sequence in Tunisia was reported on 2 December 2021. The distribution over time of the main Omicron subvariants is described in Figure 3. BA.1 was the predominant subvariant during the early phase of the study, peaking in January 2022 (*n* = 139). Subsequently, BA.2 supplanted BA.1, becoming the most frequent subvariant by March 2022 (*n* = 123). The Omicron BA.5 epidemic period began in May 2022 and reached its peak in June 2022 (*n* = 60). This lineage remained dominant throughout July. Notably, other variants, including BA.2, BA.4, and BE (BA.5-derived), circulated concomitantly with BA.5 during summer 2022. By September, BA.5 prevalence decreased, while its descendant, the BQ subvariant, rose in circulation and peaked in November (*n* = 11) of that same year. Between December 2022 and June 2023, multiple Omicron subvariants circulated, comprising recombinants such as XBB, EG, FL, and GS, alongside BA.2. Throughout summer 2023, the Omicron recombinants took over, and a peak made of XBB (*n* = 32) and EG (*n* = 20) was observed by August 2023. Their prevalence dropped by October 2023. From November 2023 onward, a decline in reported Omicron cases was observed and coincided with the emergence of the JN subvariant. In 2024, the low case count persisted, and the JN subvariant (BA.2-derived) became the main circulating one, with an apex in January 2024 (*n* = 12), alongside sporadic detection of other BA.2 derivatives.

### 3.4. Recombinant Epidemiology

**Figure 4:**
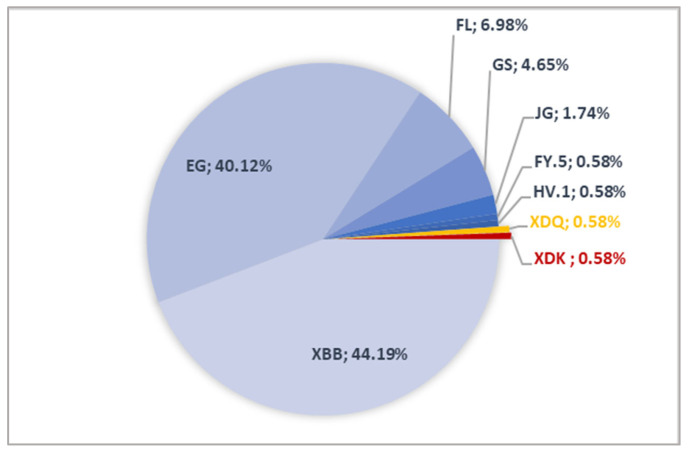
Omicron recombinant distribution among the Tunisian general population.

Within the dataset, 172 sequences were Omicron recombinants. The vast majority (*n* = 170) belonged to the XBB lineage. Among the XBB recombinants, the EG recombinant had the highest prevalence (*n* = 69), mainly represented by EG.4/EG.4.5 (*n* = 35) and EG.13 (*n* = 27), with additional cases of EG.5.1.1/EG.5.1.3 (*n* = 3) and EG.1 and EG.2 (each, *n* = 2). XBB.2.3.11 was also prevalent (*n* = 35), followed by XBB.1.5.* (*n* = 18), XBB.1.9.* (*n* = 8), and XBB.1.16.* (*n* = 6) (Figure 4). Less common recombinants included XBB.1/XBB.1.11.1 (*n* = 4) and XBB (*n* = 3) along with isolated cases of XBB.1.22.1 and XBB.1.28.1. FL was also found (*n* = 12), including FL.13 (*n* = 3), FL.1/FL.1.5.1 (*n* = 3), FL.2 (*n* = 2), and single cases of FL.10, FL.24, FL.25, and FL.4 (*n* = 1 each). Among XBB derivatives, GS.4/GS.4.1 (*n* = 8), JG.2/JG.3 (*n* = 3), and sporadic cases of FY.5 and HV.1 (*n* = 1 each) were also found. The two other Omicron recombinants were XDK (*n* = 1) and XDQ (*n* = 1). Figure 4 details the Omicron recombinant landscape in Tunisia (Figure 4).

### 3.5. Major Omicron Recombinant Mutations

**Figure 5:**
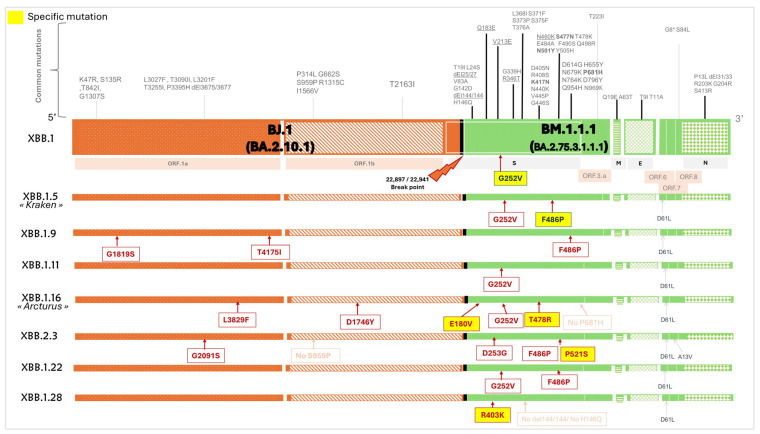
Schematic overview of the genomic structure and characteristic mutations of major Omicron XBB lineages identified in Tunisia. The top panel shows the genome organization of XBB.1 with annotated ORFs. It illustrates its origin from BJ.1 (orange stripes) and BM.1.1.1 (green checks), with a breakpoint at position 22,887–22,941 in the spike gene. The bottom panels show XBB.1 derivatives. Common mutations are shown in gray, lineage-defining mutations are highlighted in yellow, additional mutations are annotated in red, and absence of mutation is indicated in pink. Data from GISAID [16] and outbreak info (https://outbreak.info/) (accessed on 25 March 2025).

**Figure 6:**
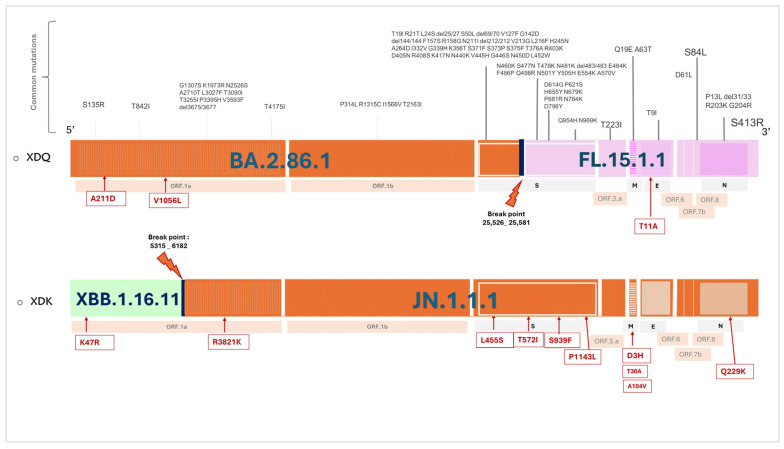
Schematic overview of the genomic structure and characteristic mutations of major Omicron XDA lineages identified in Tunisia. The top panel shows the genome organization of XDQ with annotated ORFs. It illustrates its origin from BA.2.86.1 (orange) and FL.15.1.1 (pink), with a breakpoint at position 25,528–25,581 in the spike gene. The second panel shows XDK with annotated ORFs. It illustrates its origin from XBB.1.16.11 (green) and JN.1.1.1 (orange), with a breakpoint at position 5315–6182 in the ORF.1. a. Common mutations are shown in gray; additional mutations are annotated in red. Data from GISAID [16] and outbreak info (https://outbreak.info/) (accessed on 25 March 2025).

The XBB recombinant issued from reassortment between BA.2.10.1 (BJ.1) and BA.2.75 (BM.1.1.1) with a breakpoint in S1, located around 22,897–22,941. As for the XDA recombinant, it resulted from a recombination between JN (BA.2.86) derivatives and XBB derivatives. XDK is a hybrid of XBB.1.16.11 and JN.1.1.1, with a recombination breakpoint located between nucleotide positions 5315 and 6182. Meanwhile, XDQ resulted from BA.2.86.1 and FL.15.1.1, with a recombination breakpoint between 23,605 and 23,777. Distinguishing mutations of main XBB and XDA recombinants were retrieved from Gisaid.org and related sources and are highlighted in Figure 5 and Figure 6.

### 3.6. Phylogenetic Analysis

The phylogenetic analyses were performed on the most predominant Omicron subvariants in Tunisia: BA.1, BA.2, and BA.5.

#### 3.6.1. BA.1

**Figure 7:**
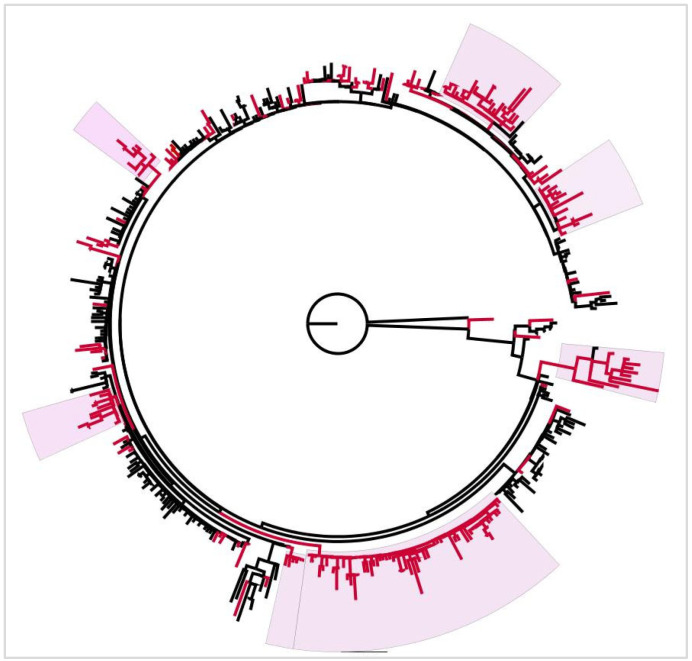
Maximum Likelihood phylogenetic tree of SARS-CoV-2 Omicron BA.1 subvariant, constructed using IQ-TREE. Red branches correspond to Tunisian sequences, and black branches correspond to worldwide sequences. Tunisian exclusive clusters are highlighted in pink. The tree is rooted using the Wuhan reference sequence (NC_045512) as an outgroup.

The phylogenetic tree of Omicron BA.1 was constructed using 223 Tunisian sequences belonging to this subvariant along with 226 global ones. The tree revealed multiple mixed clusters comprising both Tunisian and global sequences, along with several collections containing only Tunisian sequences; these collections appeared genetically independent from the global sequences (Figure 7).

#### 3.6.2. BA.2

**Figure 8:**
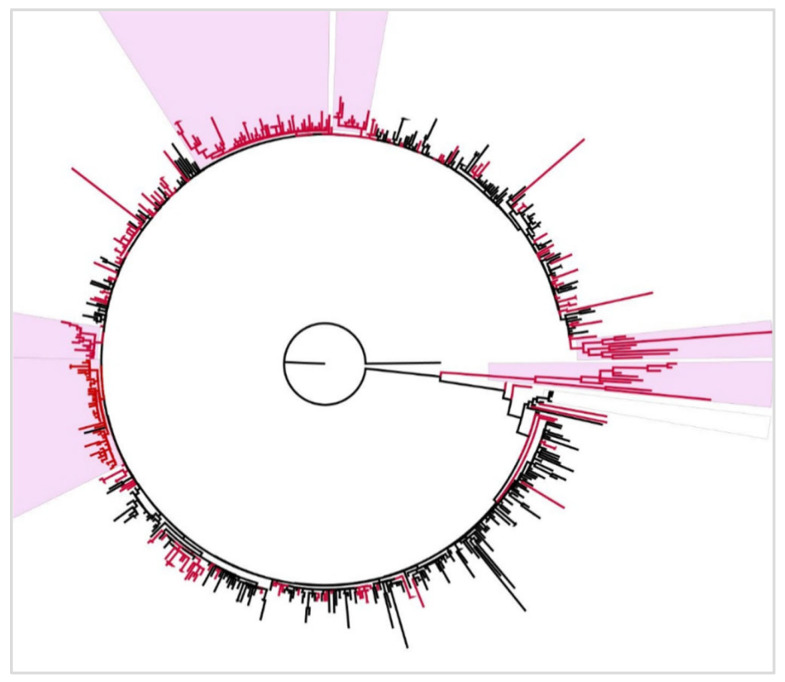
Maximum Likelihood phylogenetic tree of SARS-CoV-2 Omicron BA.2 subvariant, constructed using IQ-TREE. Red branches correspond to Tunisian sequences, and black branches correspond to worldwide sequences. Tunisian exclusive clusters are highlighted in pink. The tree is rooted using the Wuhan reference sequence (NC_045512) as an outgroup.

The phylogenetic tree of the Omicron BA.2 subvariant was constructed using 312 Tunisian sequences along with 304 global ones. The tree showed limited intermixing of Tunisian sequences within global clusters and displayed several groups composed exclusively of Tunisian sequences. Tunisian clusters appeared genetically independent from global sequences (Figure 8).

#### 3.6.3. BA.5

**Figure 9:**
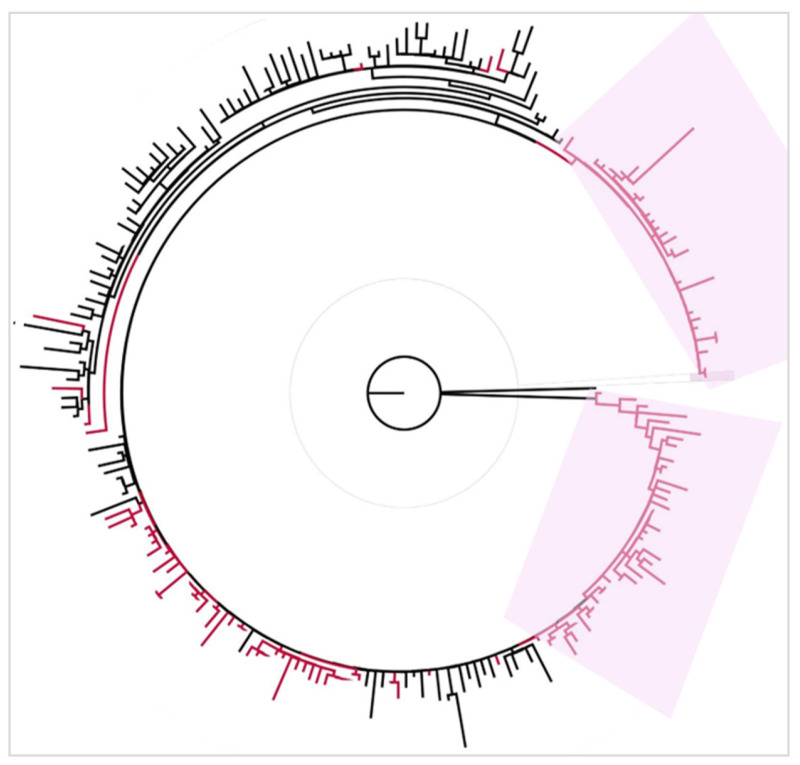
Maximum Likelihood phylogenetic tree of SARS-CoV-2 Omicron BA.5 subvariant, constructed using IQ-TREE. Red branches correspond to Tunisian sequences, and black branches correspond to worldwide sequences. Tunisian exclusive clusters are highlighted in pink. The tree is rooted using the Wuhan reference sequence (NC_045512) as an outgroup.

The phylogenetic tree of the Omicron BA.5 subvariant was constructed using 122 Tunisian and 105 global ones. The tree showed broadly gathered Tunisian sequences within well-defined clades. The remaining sequences were distributed showing intermixing with global sequences. Notably, the tree also displayed a few elongated branches, suggesting the presence of a higher genetic divergence within some sequences (Figure 9).

## 4. Discussion

The Omicron variant caused the most recent wave of COVID-19 infections and played a key role in the ongoing evolution of the SARS-CoV-2 virus [21]. According to prior research, Omicron harbors a significantly higher number of mutations compared to previous variants [8,11]. Furthermore, Omicron’s major subvariants, namely, BA.1, BA.2, BA.3, BA.4, and BA.5, showed an increased ability to escape the neutralization efficiency induced by prior vaccination or infections. Moreover, several Omicron recombinants, XBB, XBD, and XBF, have emerged and shaped this variant landscape [7]. Although Omicron has been well studied in several regions of the world, data from Tunisia were not exhaustive and covered only a few aspects of the variant epidemiology, such as its circulation among the pediatric population [14] or a case report related to a specific sub-lineage like JN.1 [22]. The present retrospective study aims to better analyze the Omicron wave and to gain a broader understanding of its dynamic in the Tunisian general population.

This study covers a 33-month period starting from 2 December 2021, the date of the first Omicron case detection in Tunisia. A diverse array of Omicron subvariants was found: BA.2, BA.1, recombinant lineages, BA.5, BA.4, and the ancestral B.1.1.529 strain, along with their respective derivatives. Notably, BA.3 was not detected, and no BA.3 sequence from Tunisia was available in GISAID up to date, supporting its total absence in the country [15]. Globally and in the USA, Muthusamy et al. reported B.1.1.529, followed by BA.2 and XBB.1, as major subvariants, while in Italy, Bergana et al. reported BA.1 as initially dominant, followed by BA.2 and BA.5 [23,24].

In early 2022, BA.1 became dominant among Tunisian isolates. This lineage contains numerous mutations, such as G339D, S371L, and N501Y, which have been associated with increased transmissibility and immune evasion [25]. The BA.1 phylogenetic analysis showed exclusive Tunisian clustering indicating local transmission, while other clusters displayed intermixing of Tunisian and worldwide sequences. This supports the occurrence of multiple importation events alongside autochthonous spread. Our findings align with previous reports in North Africa that pointed to multiple introductions of the Omicron variant likely originating from England, Scotland, and the United States [26]. By March 2022, BA.1 was supplanted by BA.2, which turned out later to be the most prevalent variant in this study. The BA.2 peak was observed during the boost immunization period (PIP3: 1 December 2021 to 3 March 2022), which is consistent with the hypothesis of increased immune evasion [27]. Additionally, prior studies have linked BA.2 evolution to host immune evasion and adaptation to epidemiological conditions [11,26]. Chatterjee et al. related BA.2’s transmissibility to the H78Y mutation, enabling its global dominance [7]. Furthermore, our study found various derivatives of BA.2, such as JN, BN, and KP. Notably, BA.2 derivatives re-emerged long after BA.2’s initial peak, and JN subvariant (BA.2.86.1 descendant) frequency began rising in January 2024, despite a significant decline in COVID-19 cases. A Brazilian study identified different JN circulation patterns: its emergence following low Severe Acute Respiratory Infection incidence, co-circulation with XBB.1, and delayed waves post-XBB.1 peaks [28]. Notably, JN.1’s S:L455S mutation, previously linked to enhanced viral fitness, may explain JN outcompeting XBB transmissibility in our study [29]. As for the BA.2 phylogenetic tree, it revealed large Tunisian clusters, attributable to the establishment of a localized evolution and a possible adaptation of the circulating strains. The independent Tunisian clades, observed in both BA.1 and BA.2 phylogenetic trees, likely reflect their heavy circulation in early 2022, a period marked by an increased economic activity, school reopening, and relaxed preventive measures. Similar exclusive genetic clusters were previously observed in the Tunisian population during the Delta wave [14].

Another major subvariant in this work was BA.5, along with its derivatives BQ and BE. In contrast, BA.4 was far less frequent, confirming its minor role in the Omicron wave in Tunisia. This aligns with global patterns where BA.5 consistently outcompeted BA.4 due to its higher effective reproduction number (R_e_) [30]. Previous studies have attributed BA.5’s strong immune evasion to mutations like L452R and F486V in addition to enhanced binding affinity and viral fitness [31]. In our study, BA.5 started peaking around May 2022, and its derivatives, BQ and BE, took over later that year. BA.5’s peak coincided with PIP4 (3 March to 1 December 2022), a period characterized by reduced population immunity and relaxed non-pharmaceutical interventions which may have facilitated sustained viral circulation and local diversification of the BA.5 lineage [27]. Phylogenetic analysis of BA.5 showed well-defined clusters, strongly suggesting in-country evolution rather than through importation events. Overall, a similar Omicron subvariant dynamic has been reported in the literature. In Japan, BA.2 was dominant from 25 April to 26 June 2022, followed by BA.5 from 18 July to 25 September 2022 [32], and, in India, second-generation BA.2 lineages circulated extensively during summer 2022 but failed to spread widely in regions where BA.5 was dominant [30].

Another prominent group identified in the present study was the Omicron recombinants group, nearly all belonging to the XBB sub-lineage. Focosi and Maggi documented the identification of over 75 recombinants so far among SARS-CoV-2, ranging from XA to XY [33]. Based on the literature, the high genetic divergence during the Omicron era, along with broad diversity and regional variations, were favorable for co-infection with distinct viral strains, a prerequisite for recombination [34]. The XBB subvariant, as evidenced previously, arose from BA.2.10.1 (BJ.1) and BA.2.75 (BM.1.1.1) and acquired critical mutations like Y144del, V83A, N460K, and F486S. Tamura et al. linked those mutations to an increased viral fitness and immune evasion. They also described XBB as the first SARS-CoV-2 variant to increase its fitness through recombination rather than substitution [35]. In Tunisia, recombinants started emerging in late 2022, similarly to what was reported in Singapore [36], and went on to account for nearly 20% of all Omicron cases. Subsequently, two successive XBB-driven waves were observed during spring and summer 2023. The shift towards BQ and XBB at the time was attributed to their higher R_e_ compared to predecessor BA.5 [35], and this period was referred to as the ‘variant soup,’ as it was marked by the co-circulation of multiple subvariants [37]. Global trends, where XBB outcompeted earlier Omicron subvariants, supported recombination as a key driver of the virus’s evolution [12]. In the present study, the EG lineage (XBB.1.9.2 descendant) was the predominant recombinant, including EG.4, EG.4.5, and EG.13. While in the US, EG.5 (‘Eris’) caused 20% of the infections in August 2023, its detection was rare in our cohort [38]. Other frequent recombinants were detected in our study: XBB.2.3.11, XBB.1.5, XBB.1.9, and XBB.1.16, along with XBB-derived lineages like FL, GS, and JG. FL.1.5.1’s detection is interesting given it was highly reported in the US [39]. In addition to XBB, rare detection of JN.1-derived recombinants (XDK and XDQ) was noted. These recombinants carried the 17MPLF insertion, previously documented to enhance spike compactness and ACE2 binding [40].

It is worth noting that random sampling of the global sequences in the phylogenetic analysis may not capture full subvariant diversity, limiting comparative analyses, and that the absence of clinical data hinders correlation with epidemiological trends.

## 5. Conclusions

The Omicron era in Tunisia was marked by a high diversity of subvariants, shaped by importation events as well as localized evolution. BA.2 was the most prevalent subvariant, and its recombinant descendant, XBB, played a significant role in defining the Omicron wave in Tunisia. The gradual shift toward BA.2 derivatives and recombinants in the wave’s later stages highlights the virus’s continuous adaptation to host immunity. These findings emphasize the critical importance of sustained genomic surveillance integrated with epidemiological data to anticipate and mitigate the impact of future recombinant-driven outbreaks.

## Data Availability

The original contributions presented in this study are included in the article/Supplementary Material. Further inquiries can be directed to the corresponding author.

## Supplementary Materials

The following supporting information can be downloaded at: https://www.mdpi.com/article/10.3390/microorganisms13092162/s1, Table S1: Accession ID and virus name and related details of complete genome SARS-CoV-2 Tunisian sequences analyzed in this study and retrieved from GISAID. Table S2: Accession ID, submitters, and geographical origin of complete genome SARS-CoV-2 sequences used to build phylogenetic trees and retrieved from GenBank.

## Abbreviations

The following abbreviations are used in this manuscript:

SARS-CoV-2	Severe Acute Respiratory Syndrome Coronavirus 2
WHO	World Health Organization
COVID-19	Coronavirus Disease 2019
VOC	Variant of Concern
VOI	Variant of Interest
VUM	Variant Under Monitoring
Pango	Phylogenetic Assignment of Named Global Outbreak Lineages
ACE2	Angiotensin-Converting Enzyme 2
RBD	Receptor Binding Domain
FCS	Furin-like Cleavage Site
GISAID	Global Initiative on Sharing All Influenza Data
NGS	Next-Generation Sequencing
IZS-Te	Istituto Zooprofilattico Sperimentale dell’Abruzzo e del Molise, Teramo
NCBI	National Center for Biotechnology Information
MAFFT	Multiple Alignment using Fast Fourier Transform
IQ-TREE	Efficient Phylogenetic Software for Maximum Likelihood Analysis
Re	Effective Reproduction Number
NPIs	Non-Pharmaceutical Interventions
PIs	Pharmaceutical Interventions
PIP	Pharmaceutical Intervention Period

## References

[1] Cucinotta D., Vanelli M. (2020). WHO Declares COVID-19 a Pandemic. Acta Biomed..

[2] World Health Organization (2024). COVID-19 Cases|WHO COVID-19 Dashboard. https://data.who.int/dashboards/covid19/cases.

[3] Wang C., Horby P.W., Hayden F.G., Gao G.F. (2020). A Novel Coronavirus Outbreak of Global Health Concern. Lancet.

[4] Rouzine I.M. (2025). Evolutionary mechanisms of the emergence of the variants of concern of SARS-CoV-2. Viruses.

[5] World Health Organization (2025). Tracking SARS-CoV-2 Variants. https://www.who.int/activities/tracking-SARS-CoV-2-variants.

[6] Rambaut A., Holmes E.C., O’Toole Á., Hill V., McCrone J.T., Ruis C., du Plessis L., Pybus O.G. (2020). A Dynamic Nomenclature Proposal for SARS-CoV-2 Lineages to Assist Genomic Epidemiology. Nat. Microbiol..

[7] Chatterjee S., Bhattacharya M., Nag S., Dhama K., Chakraborty C. (2023). A Detailed Overview of SARS-CoV-2 Omicron: Its Sub-Variants, Mutations and Pathophysiology, Clinical Characteristics, Immunological Landscape, Immune Escape, and Therapies. Viruses.

[8] Das S., Samanta S., Banerjee J., Pal A., Giri B., Kar S.S., Dash S.K. (2022). Is Omicron the End of Pandemic or Start of a New Innings?. Travel Med. Infect. Dis..

[9] He X., Hong W., Pan X., Lu G., Wei X. (2021). SARS-CoV-2 Omicron Variant: Characteristics and Prevention. MedComm.

[10] Shah M., Woo H.G. (2022). Omicron: A Heavily Mutated SARS-CoV-2 Variant Exhibits Stronger Binding to ACE2 and Potently Escapes Approved COVID-19 Therapeutic Antibodies. Front. Immunol..

[11] Wang L., Møhlenberg M., Wang P., Zhou H. (2023). Immune Evasion of Neutralizing Antibodies by SARS-CoV-2 Omicron. Cytokine Growth Factor Rev..

[12] Thakur P., Thakur V., Kumar P., Patel S.K.S. (2022). Emergence of Novel Omicron Hybrid Variants: BA(x), XE, XD, XF More Than Just Alphabets. Int. J. Surg..

[13] Chouikha A., Fares W., Laamari A., Haddad-Boubaker S., Belaiba Z., Ghedira K., Kammoun Rebai W., Ayouni K., Khedhiri M., Ben Halima S. (2022). Molecular epidemiology of SARS-CoV-2 in Tunisia (North Africa) through several successive waves of COVID-19. Viruses.

[14] Haddad-Boubaker S., Arbi M., Souiai O., Chouikha A., Fares W., Edington K., Sims S., Camma C., Lorusso A., Diagne M.M. (2023). The Delta Variant Wave in Tunisia: Genetic Diversity, Spatio-Temporal Distribution, and Evidence of the Spread of a Divergent AY.122 Sub-Lineage. Front. Public Health.

[15] Khemiri H., Mangone I., Gdoura M., Mefteh K., Chouikha A., Fares W., Lorusso A., Ancora M., Pasquale A.D., Cammà C. (2024). Dynamic of SARS-CoV-2 Variants Circulation in Tunisian Pediatric Population, During Successive Waves, from March 2020 to September 2022. Virus Res..

[16] (2025). GISAID Initiative. https://www.epicov.org/epi3/frontend#d95e5.

[17] (2004). NCBI VirusBethesda (MD): National Library of Medicine (US), National Center for Biotechnology Information. https://www.ncbi.nlm.nih.gov/labs/virus/vssi/#/.

[18] Katoh K., Rozewicki J., Yamada K.D. (2019). MAFFT Online Service: Multiple Sequence Alignment, Interactive Sequence Choice and Visualization. Brief. Bioinform..

[19] Minh B.Q., Schmidt H.A., Chernomor O., Schrempf D., Woodhams M.D., von Haeseler A., Lanfear R. (2020). IQ-TREE 2: New Models and Efficient Methods for Phylogenetic Inference in the Genomic Era. Mol. Biol. Evol..

[20] Rambaut A. (2010). FigTree v1.3.1. Institute of Evolutionary Biology, University of Edinburgh, Edinburgh. http://tree.bio.ed.ac.uk/software/figtree/.

[21] González-Candelas F., Shaw M.A., Phan T., Kulkarni-Kale U., Paraskevis D., Pybus O.G., Kraemer M.U.G. (2021). One Year into the Pandemic: Short-Term Evolution of SARS-CoV-2 and Emergence of New Lineages. Infect. Genet. Evol..

[22] Hamzaoui Z., Ferjani S., Kanzari L., Ben Ali R., Charaa L., Landolsi I., Medini I., Chammam S., Abid S., Ferjani A. (2024). Unveiling the Emergence of SARS-CoV-2 JN.1 Sub-Variant: Insights from the First Cases at Charles Nicolle Hospital, Tunisia. Acta Microbiol. Immunol. Hung..

[23] Muthusami R., Saritha K. (2023). Exploratory Analysis of SARS-CoV-2 Omicron Variant and Its Subvariant Propagation: Global Predominance of BA.1, BA.2, BA.5, BE.1, and BQ.1. Proc. Indian Natl. Sci. Acad. Part A Phys. Sci..

[24] Bergna A., Lai A., Sagradi F., Menzo S., Mancini N., Bruzzone B., Rusconi S., Marchegiani G., Clementi N., Francisci D. (2025). Genomic Epidemiology of the Main SARS-CoV-2 Variants Circulating in Italy During the Omicron Era. J. Med. Virol..

[25] Kumar S., Thambiraja T.S., Karuppanan K., Subramaniam G. (2022). Omicron and Delta Variant of SARS-CoV-2: A Comparative Computational Study of Spike Protein. J. Med. Virol..

[26] Menasria T., Aguilera M. (2022). Genomic Diversity of SARS-CoV-2 in Algeria and North African Countries: What We Know So Far and What We Expect?. Microorganisms.

[27] Abroug H., Ouanes-Besbes L., Dachraoui F., Ouanes I., Addad F., Hdiji A., Ben Romdhane H. (2024). Impact of Pharmaceutical and Non-Pharmaceutical Interventions on COVID-19 in Tunisia. BMC Public Health.

[28] Tort L.F.L., Naveca M.M., Nascimento V.A., Souza V.C., Fernandes L.T., Gomes K.R., Costa A.J. (2024). SARS-CoV-2 Omicron XBB Infections Boost Cross-Variant Neutralizing Antibodies, Potentially Explaining the Observed Delay of the JN.1 Wave in Some Brazilian Regions. IJID Reg..

[29] Li Z., Zhang Y., Lu J., Chen Y., Huang X., Zhang L., Wang Q. (2024). Molecular Epidemiology and Population Immunity of SARS-CoV-2 in Guangdong (2022–2023) Following a Pivotal Shift in the Pandemic. Nat. Commun..

[30] Yajima H., Ito J., Ueno T., Sato K. (2024). Molecular and Structural Insights into SARS-CoV-2 Evolution: From BA.2 to XBB Subvariants. mBio.

[31] Cao Y., Wang J., Jian F., Xiao T., Song W., Yisimayi A., Huang W., Li Q., Wang P., An R. (2022). BA.2.12.1, BA.4 and BA.5 Escape Antibodies Elicited by Omicron Infection. Nature.

[32] Nakakubo S., Kishida N., Okuda K., Kamada K., Iwama M., Suzuki M., Yokota I., Ito Y.M., Nasuhara Y., Boucher R.C. (2023). Associations of COVID-19 Symptoms with Omicron Subvariants BA.2 and BA.5, Host Status, and Clinical Outcomes: A registry-based observational study in Sapporo, Japan. Lancet Infect. Dis..

[33] Focosi D., Maggi F. (2022). Recombination in Coronaviruses, with a Focus on SARS-CoV-2. Viruses.

[34] Wang Q., Iketani S., Li Z., Liu L., Guo Y., Huang Y., Bowen A.D., Liu M., Wang M., Yu J. (2023). Alarming antibody evasion properties of rising SARS-CoV-2 BQ and XBB subvariants. Cell.

[35] Tamura T., Ito J., Uriu K., Zahradnik J., Kida I., Nasser H., Shofa M., Oda Y., Lytras S., Nao N. (2023). Virological characteristics of the SARS-CoV-2 XBB variant derived from recombination of two Omicron subvariants. Nat. Commun..

[36] Chia T.R.T., Young B.E., Chia P.Y. The Omicron-Transformer: Rise of the Subvariants in the Age of Vaccines. Annals Singapore. https://annals.edu.sg/the-omicron-transformer-rise-of-the-subvariants-in-the-age-of-vaccines/.

[37] Focosi D., Quiroga R., McConnell S., Johnson M.C., Casadevall A. (2023). Convergent evolution in SARS-CoV-2 spike creates a variant soup from which new COVID-19 waves emerge. Int. J. Mol. Sci..

[38] Sil D., Gautam S., Saxena S., Joshi S., Kumar D., Mehta A., Jindal P., Sharma S., Pandey P., Diksha Comprehensive Analysis of Omicron Subvariants: EG.5 Rise, Vaccination Strategies, and Global Impact. EurekaSelect. https://www.eurekaselect.com/article/140269.

[39] Şimşek-Yavuz S. (2023). COVID-19: An Update on Epidemiology, Prevention and Treatment, September-2023. Infect. Dis. Clin. Microbiol..

[40] Chakraborty A.K. (2024). Rapid Worldwide Spread of 17MPLF Spike Insertion Mutants (JN.1-JN.1.25, KP.1, KP.2, KQ.1, KR.1, XDD, XDP, XDK, XDQ Subvariants) of Omicron Coronaviruses and Spike Gene 5′-End Sequencing Problem. SciTe.ai. ResearchSquare.

